# Effects of different colloid infusions on ROTEM and Multiplate during elective brain tumour neurosurgery

**DOI:** 10.1186/s13741-015-0019-7

**Published:** 2015-09-29

**Authors:** N. Li, S. Statkevicius, B. Asgeirsson, U. Schött

**Affiliations:** Department of Medicine, Växjö County Hospital, Växjö, Sweden; Department of Anaesthesia and Intensive Care, Lund University and Skane University Hospital, Lund, S-22185 Sweden

**Keywords:** Thromboelastography, Platelet aggregation, Albumin, Hydroxyethyl starch, Neurosurgery

## Abstract

**Background:**

The European Medicines Agency does not recommend the use of hydroxyethyl starch-based volume replacement solutions in critically ill patients due to an increased risk of renal failure. However, this recommendation is questionable for its perioperative use. Several recent randomised controlled studies do not indicate a risk for renal failure—not even after high-risk surgery. Human albumin is used in our neurointensive care unit as a part of the “Lund concept” of brain injury resuscitation, and albumin has been introduced in elective neurosurgery instead of starch. The aim of our prospective unblinded observational cohort study was to compare the degree of dilutive coagulopathy after albumin and starch intra-operative fluid therapy.

**Methods:**

Thirty-nine patients undergoing elective brain tumour surgery with craniotomy received either 130/0.42 hydroxyethyl starch or 5 % albumin infusions. The first 18 patients received starch, whereas the rest received albumin. Rotational thromboelastometry with ROTEM and platelet aggregometry with Multiplate were performed before surgery, after the first and second consecutive colloid infusions (250/500 ml albumin or 500/1000 ml starch) and at the end of surgery.

**Results:**

Both intra- and inter-group comparisons showed more deranged ROTEM parameters after the higher doses of starch. Multiplate detected changes only in the albumin group after 500-ml infusion. Blood los did not differ between groups, nor did haemoglobin preoperatively or at end of surgery. Lower volumes of albumin were required to maintain stable intra-operative haemodynamic parameters; 250/500 ml albumin corresponded to 500/1000 ml starch.

**Conclusions:**

Hydroxyethyl starch affected coagulation at lower volumes, with a more prominent effect on clot structure at the end of surgery, corroborating previous research. Only albumin decreased platelet aggregation, and 5 % albumin had a more potential volume effect than 130/0.42 hydroxyethyl starch.

## Background

In brain tumour neurosurgery, intra- and post-operative bleeding in brain tumour resection can be linked to the vascularity of the tumours, tumour size and localization (Goh et al. 1997). Most of the losses will be surgical, and as the field is open, direct haemostasis with cautery or topical coagulants is more relevant. Post-operative hematomas are rare but may be more related to a coagulopathy than a surgical bleed. During surgery, different fluids are used to replace blood loss and maintain arterial pressure, including crystalloids and blood products, and during the last few decades, synthetic colloids as well. However, all of these fluids are associated with adverse effects, and an ideal fluid for haemodynamic stabilisation and resuscitation has yet to be found.

Hydroxyethyl starches (HESs) are synthetic colloids with different molecular weights and substitutions that are used for their plasma-expanding effects. In recent years, several studies have highlighted the adverse effects of this colloid, particularly the risk of acute renal failure (Zarychanski et al. 2013), allergic skin manifestations and hypocoagulability following infusion. HES has been shown to impair clot strength, platelet function and increase fibrinolysis to an extent that cannot be explained by haemodilution alone (Levi & Jonge 2007). HES infusions decrease plasma levels of fibrinogen and several coagulation factors, leading to weaker and smaller clots (Fenger-Eriksen et al. 2009). It can also decrease circulating levels of von Willebrand’s factor (F), thus impairing platelet function (de Jonge et al. 2001).

In Europe, market authorisations for HES have been suspended due to these findings (Agency EM. PRAC recommends suspending marketing authorisations for infusion solutions containing hydroxyethyl starch: European Medicines Agency & [cited 2013 Jun 14]). However, the clinical perioperative implications of these findings are still uncertain for stable patients undergoing elective surgery (Moral et al. 2013)*.* Human albumin (HA) is an alternative fluid that has recently been replacing HES in neurosurgery at Lund University Hospital. Albumin is used by us in accordance with the “Lund concept” of brain injury resuscitation (Grande 2011), and for us, it was natural to replace HES with HA. However, in large meta-analyses on the effectiveness of HA on patient mortality and morbidity, no significant benefits have been shown when comparing HA to synthetic colloids or crystalloids (Perel et al. 2013; Roberts et al. 2011). HA may have lesser impact on coagulation compared to synthetic colloids (Niemi et al. 2006), which is a desirable quality for perioperative use. The coagulopathy caused by HA also seems to be easily reversed by fibrinogen and FXIII concentrate (Winstedt et al. 2013), making it a potentially better fluid for patients that might already suffer from a coagulopathy or risk of developing one.

Routine clinical laboratory analyses of coagulation, such as activated partial thromboplastin time (aPTT) and prothrombin time (PT), are plasma-based and might not correctly predict a clinical coagulopathy. Whole blood viscoelastic methods, such as thromboelastometry (e.g. ROTEM®) and whole blood-aggregometry (e.g. Multiplate®), have gained recognition as alternative methods. Thromboelastometry is able to assess global haemostatic functions, and platelet aggregometry assesses platelet function in response to different reagents. These systems have already been introduced clinically as point-of-care methods for quickly determining bleeding risk and helping to guide transfusions (Shore-Lesserson et al. 1999). They have also been used to study haemostasis in different critical care situations, such as trauma (Solomon et al. 2011) and sepsis (Brenner et al. 2012). ROTEM is known to detect colloid-induced coagulopathy (Fenger-Eriksen et al. 2009), even at low levels of dilution (Tynngård et al. 2014).

The purpose of this study was to compare the effects on coagulation of 5 % HA and HES 130/0.42 in elective brain tumour neurosurgical patients. Our aim was to investigate whether HA had a more favourable effect on coagulation and platelet function, assessed by a viscoelastic method and a platelet aggregometry method (ROTEM and Multiplate, respectively). Our hypothesis was that HA infusions would induce less hypocoagulability than HES, as seen on ROTEM and Multiplate.

## Methods

This study was performed as a prospective unblinded observational cohort study with two colloid fluid regimens. During 2013 and 2014, our fluid regimen for haemodynamic stabilisation and initial blood-loss substitution during elective brain tumour resection was changed from HES to 5 % HA. We had studied 18 consecutive patients with HES according to a protocol, and our initial aim was to include more patients with HES and the combined testing with ROTEM and Multiplate when the department replaced HES with HA.

We therefore decided to continue the protocol with HA and compare its effects on ROTEM and Multiplate with the data from the previous HES-patients. According to a power analysis from an in vitro study (Winstedt et al. 2013), >15 patients in each group would give a statistical power of 0.8 at a significance level of *p* < 0.05, defined by detected differences in FIBTEM-MCF (see below), as this is the best predictive ROTEM parameter for dilutive coagulopathy, correlating with fibrinogen; the first coagulation factor to reach critical low levels during haemodilution (Winstedt et al. 2013).

General ethical approval was obtained from the Regional Ethical Review Board (Lund, Protocol DNR 2012/482) for monitoring neurosurgery patients with ROTEM and Multiplate. Signed consent was received from all patients in the two test groups. All patients were over 18 years.

No patients with a known haemostatic disturbance, anticoagulants, antiplatelet drugs (also including aspirin/nonsteroidal anti-inflammatory drugs), abnormal preoperative coagulation analysis (aPTT/PT, platelet count), known renal impairment or increased plasma-creatinine level were included.

Anaesthesia was induced and maintained with propofol (Diprivan®; AstraZeneca, Sweden) and remifentanil (Ultiva®, GlaxoSmithKline, Sweden). Intubation was facilitated with rocuronium (Esmeron®; MSD, USA) (0.5–0.8 mg/kg), and ventilation was maintained with positive pressure ventilation in a circle system. Minute ventilation was adjusted to maintain normocapnia (PaCO2 of 4.5–5.5 kPa).

After induction, a radial arterial catheter was inserted for continuous measurement of blood pressure and for collection of blood samples, and a bladder catheter was inserted for hourly measurement of diuresis.

All patients received thromboprophylaxis with mechanical calf compression (Kendall SCD™ Express Sleeves; Covidien, USA) during surgery and postoperatively for 24 h.

Normothermia during the surgery was maintained with a Bair Hugger™ (3 M, St. Paul, USA) and oesophageal temperature monitoring.

During this study, there was no intervention on our part in the transfusion/infusion protocols. The crystalloid/colloid infusions and transfusion of blood components were solely determined by the anaesthetist in charge, based on a standard protocol from the anaesthesia department (see below) and were not affected by the study protocol or the ROTEM/Multiplate test results.

Standard Lund departmental protocol for perioperative fluids during neurosurgery: After the induction of anaesthesia, a basal infusion of 1.5–2.0 ml/kg/h of saline (NaCl 0.9 %; B. Braun Medical AB) was started. Initial bleeding up to 200–300 ml was substituted with saline (2–3 ml per 1 ml of bleeding). The HES group received hydroxyethyl starch 130/0.42 in sodium chloride for maintaining mean arterial pressure of >60–65 mm Hg, systolic blood pressure >90 mmHg, pulse pressure variation (PPV) <12 mmHg and replacing bleeding of >200–300 ml (see above) (1–2 ml HES per 1 ml of bleeding) (HES; Venofundin® 60 mg/ml hydroxyethyl starch; MW 130 kDa; substitution 0.42; B. Braun Medical AG, Germany). HES was thus also used to compensate for the haemodynamic effects of anaesthesia. HES was restricted to 1000 ml by the departmental protocol. The HA group was given 5 % human albumin (Albumin; CSL Behring, Germany) at 1–2 ml HA per 1 ml of bleeding of >200–300 ml (see above). HA was also used to compensate for the haemodynamic effects of anaesthesia as for HES. Albumin was restricted to 500 ml by the departmental protocol.

According to departmental protocol, packed red blood cells (PRBCs) are to be administered when the concentration of haemoglobin reaches <95–100 gram/L, and blood loss of more than 30 % of the calculated blood volume is substituted with PRBCs, fresh-frozen plasma (FFP) and platelet concentrates (PC).

Arterial blood was sampled from radial arterial catheters with a continuous sodium chloride flush system with no heparin. The blood samples were collected in 2.7-ml citrated plastic vacuum tubes (3.2 % citrate; BD Vacutainer systems, UK) for ROTEM and in 3.0-ml hirudin tubes (Dynabyte GmbH, Germany) for Multiplate analysis. Blood sampling was performed before surgical incision, after every colloid unit (i.e. after every 250 ml HA or 500 ml HES infusion) and at the end of surgery; altogether, three or four samples were collected per patient. Blood loss during surgery was evaluated from suction reservoir and swabs.

Arterial blood gases with haemoglobin, electrolyte, lactate and glucose levels were analysed before the beginning of surgery and during surgery every 1–2 h (Radiometer ABL800 FLEX; Radiometer, Denmark).

Rotational thromboelastometry (ROTEM®; TEM Innovations GmbH, Germany) measures the coagulation initiation, amplification and propagation kinetics of whole blood. It uses a cup with a rotating pin, whose movement is impeded as blood coagulates inside the cup. The impedance reflects clot firmness and is plotted graphically against time. Analysis was carried out within 1 h from blood sampling, during which time the samples were kept at 37 °C. The following two assays were run on each sample: EXTEM and FIBTEM. EXTEM measures coagulation activated by tissue factor (extrinsic pathway), while the FIBTEM assay includes a platelet inhibitor (cytochalasin D), thus measuring only fibrinogen activity. For EXTEM, the following parameters are assessed for each analysis (normal range within brackets): clotting time/CT (42–74 s), clot formation time/CFT (46–148 s), alpha-angle/AA (63–81°) and maximum clot firmness/MCF (63–81 mm). For FIBTEM, only MCF (9–25 mm) was analysed. The normal ranges used were established in a multi-centre study (Lang et al. 2005).

Multiple electrode aggregometry (Multiplate®; Roche Diagnostics, Basel, Switzerland) measures platelet aggregation in whole blood using electrical resistance between two electrodes. As aggregation occurs, the increasing impedance is plotted against time, and the area under the curve (AUC) is a measure of platelet function. The blood samples were kept at room temperature for 30–40 min before analysis, which was done at 37 °C. The following two test assays were performed for each sample: platelet aggregation in response to adenosine diphosphate (ADP-test, AUC reference range 57–113) and thrombin receptor-activating peptide (TRAP-test, AUC reference range 84–128). For statistical analysis of the data, both inter- and intra-group comparisons were made. Normal distribution was not assumed (non-parametric data). Samples after first colloid infusion, after second colloid infusion and at the end of surgery were tested against the preoperative sample using the Wilcoxon signed-rank test for paired samples. All ROTEM and Multiplate values in one test group were also compared to the corresponding values in the other test group, using the Mann-Whitney U test for unpaired samples. The level of significance was set to *p* < 0.017 after correction in accordance to Bonferroni, in order to decrease the risk of type I errors due to multiple comparisons. Results are presented as boxplots showing median and interquartile range, with min-max whiskers and + signs identifying mean values.

## Results

In total, 18 patients were included in the HES group and 21 patients in the HA group during a 5-month period. The demographic, bleeding and transfusion/infusion data for these patients are shown in Table 1. Six patients received erythrocyte transfusions, one patient received platelet transfusions and one patient received plasma transfusions intra-operatively. There were no differences in median haemoglobin concentration at the end of surgery between the groups (Table 1). The decrease in haemoglobin preoperative-postoperative was the same in both groups.

**Table 1:** Demographic and bleeding data for patients receiving 5 % human albumin (HA) or hydroxyethyl starch 130/0.4 (HES) during elective neurosurgery up to the end of surgery (eos)

	HES (*n* = 18)	HA (*n* = 21)
Gender (male/female)	10/8	7/14
Median age (min-max)	51(21–86)	54(20–73)
Blood loss (*n*)	100–499 ml (7)	100–499 ml (8)
	500–999 ml (4)	500–999 ml (3)
	1000–1999 ml (5)	1000–1999 ml (4)
	≥2000 ml (1)	≥2000 ml (0)
Total colloid volume received (*n*)	500 ml (5)	250 ml (10)
	1000 ml (13)	500 ml (11)
Total NaCl volume received (*n*)	2000 ml (15)	2000 ml (16)
	2500 ml (2)	2500 ml (5)
	3000 ml (2)	
Median preoperative Hb (min-max)	128 g/L (99–148)	127 (97–144)
Median eos Hb (min-max)	116 g/L (96–122)	113 g/L (94–144)

### Intra-group comparisons

**Fig. 1:**
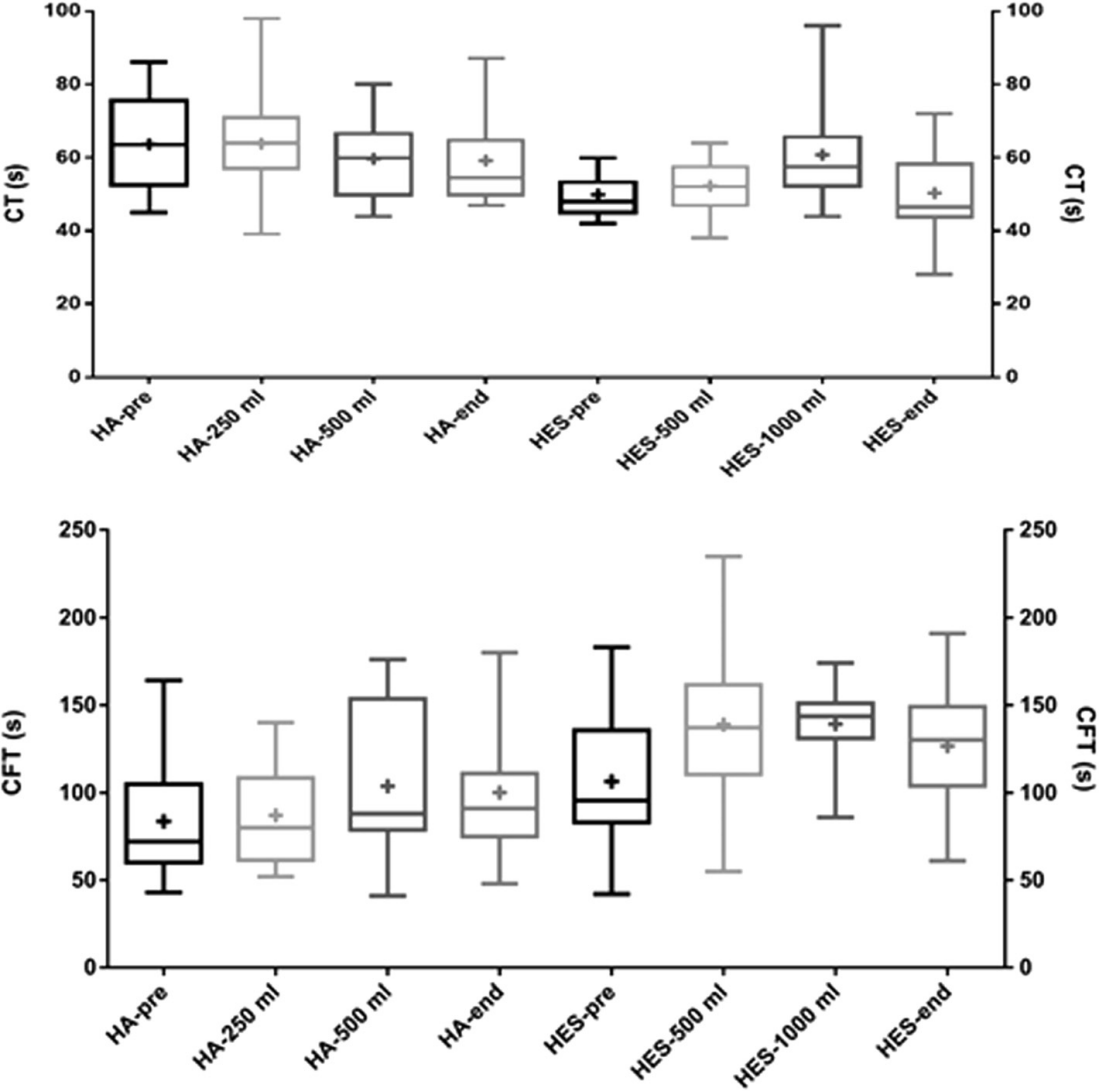
ROTEM CT and CFT values of patients undergoing elective brain tumour neurosurgery. Blood samples were taken before the start of surgery, after receiving colloid infusions (human albumin (HA) or hydroxyethyl starch (HES) 130/0.4 and at the end of surgery. *Boxplots* showing median and interquartile range with min-max whiskers and + signs identifying mean values

**Fig. 2:**
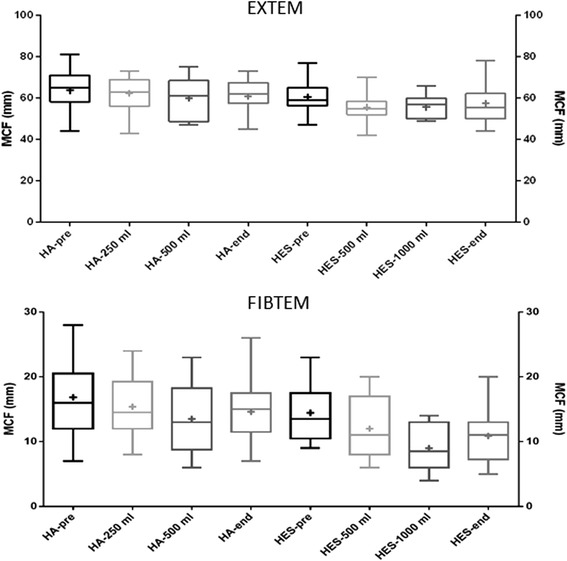
ROTEM EXTEM-MCF and FIBTEM-MCF values of patients undergoing elective brain tumour neurosurgery. Blood samples were taken before the start of surgery, after receiving colloid infusions (human albumin (HA) or hydroxyethyl starch (HES) 130/0.4 and at the end of surgery. *Boxplots* showing median and interquartile range with min-max whiskers and + signs identifying mean values

**Fig. 3:**
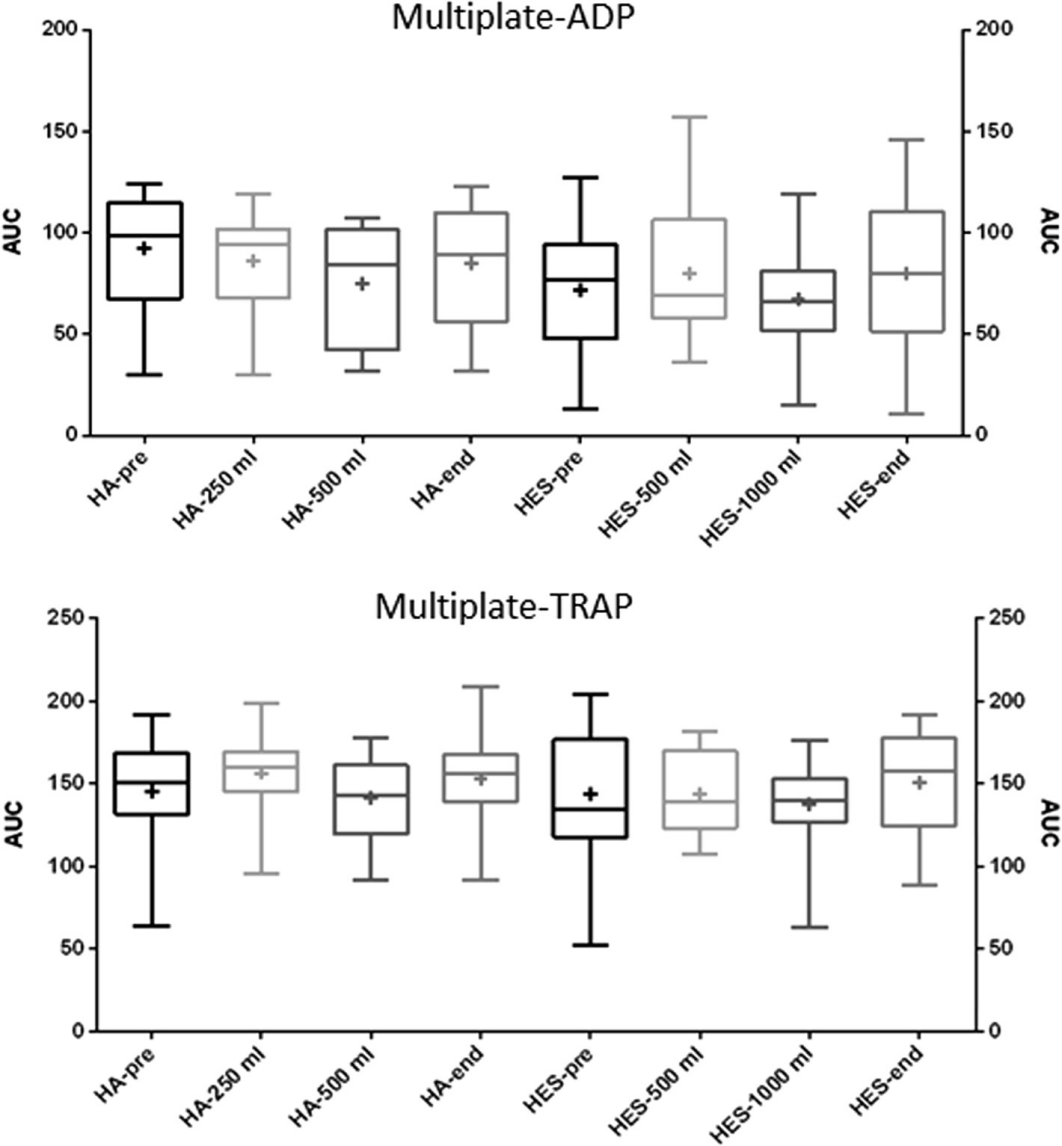
Multiplate ADP and TRAP AUC values of patients undergoing elective brain tumour neurosurgery. Blood samples were taken before the start of surgery, after receiving colloid infusions (human albumin (HA) or hydroxyethyl starch (HES) 130/0.4 and at the end of surgery. *Boxplots* showing median and interquartile range with min-max whiskers and + signs identifying mean values

Results from each sampling occasion were compared to the preoperative results. In the HA group, the most significant differences were detected after the second HA infusion, as well as at the end of surgery (Tables 2 and 3 and Figs. 1, 2 and 3). After 250 ml HA, ROTEM only showed a significant decrease in FIBTEM-MCF. After 500 ml HA, CFT, AA and EXTEM-MCF also differed as compared to pre-surgery levels. At the end of surgery, changes were detected in all ROTEM parameters except for CT. Multiplate detected changes in the ADP parameter only after 500 ml HA. No other significant changes could be shown in ADP or TRAP parameters. In the HES group, significant differences could be detected already after the first infusion (Table 2 and 3 and Figs. 1, 2 and 3). After 500 ml HES, CFT was markedly prolonged, as seen in Fig. 1. AA and EXTEM-MCF both decreased (Fig. 2 (AA not shown)). Only after 1000 ml HES was CT prolonged (Tables 2 and 3 and Fig. 1). Additionally, changes could be seen in the CFT, AA and FIBTEM-MCF parameters after 1000 ml HES, as well as after surgery (Tables 2 and 3). Multiplate did not detect any significant differences within the HES group (Fig. 3).

**Table 2:** ROTEM and Multiplate parameters (median values, first and third quartiles within brackets) of patients receiving hydroxyethyl starch 130/0.4 (HES) during brain tumour neurosurgery (*n* = 18), blood samples taken at different times during surgery

	Pre-surgery	After 500 ml HES	After 1000 ml HES	End of surgery
CT (s)	48 (45–53)	52 (47–57)	58 (54–63)	47 (44–56)
CFT (s)	96 (84–131)	137 (115–145)	144 (134–148)	130 (110–144)
AA (°)	72 (67–73)	64 (62–65)	63 (61–65)	65 (62–68)
MCF (mm)	59 (57–63)	55 (52–58)	57 (50–58)	56 (51–62)
FIBTEM-MCF (mm)	14 (11–17)	12 (8–18)	9 (6–12)	11 (8–13)
Multiplate-ADP(AUC)	77 (51–89)	69 (58–101)	66 (53–79)	80 (53–102)
Multiplate-TRAP(AUC)	135 (120–171)	139 (123–169)	140 (128–152)	158 (128–177)

**Table 3:** The *p* values when comparing ROTEM and Multiplate parameters of brain tumour neurosurgery patients after colloid infusions before surgery. Results after each colloid infusion (250 ml for HA and 500 ml for HES) as well as at the end of surgery were compared to pre-operative results using Wilcoxon’s signed ranked test

	CT (s)	CFT (s)	AA (°)	EXTEM-MCF (mm)	FIBTEM-MCF (mm)	Multiplate ADP(AUC)	Multiplate TRAP(AUC)
HA 250 ml	ns	ns	ns	ns	0.0015 (**)	ns	ns
HA 500 ml	ns	0.0098 (**)	0.0156 (*)	0.0039 (**)	0.0039 (**)	0.0020 (*)	ns
HA end of surgery	ns	0.0011 (**)	0.0012 (**)	0.0098 (**)	0.0042 (**)	ns	ns
HES 500 ml	ns	<0.0001 (****)	0.0001 (***)	0.0005 (***)	ns	ns	ns
HES 1000 ml	0.0156 (*)	0.0020 (**)	0.0039 (**)	ns	0.0020 (**)	ns	ns
HES end of surgery	ns	0.0027 (**)	0.0015 (**)	ns	0.0043 (**)	ns	ns

Significance level was set at *p* < 0.017, **p < 0.01, ***p < 0.001 and ****p < 0.0001

### Inter-group comparisons

When comparing the two test groups against each other, each parameter was compared to the corresponding one in the other test group. Most of the changes occurred after the first dose of colloid. Before the start of surgery, only the CT parameter differed between the groups (*p* = 0.0001), with a lower median CT in the HES group. After the first colloid infusion, CT was still significantly shorter in the HES group (*p* = 0.0014), while CFT was longer (*p* = 0.0002). EXTEM-MCF were lower (*p* = 0.001 and *p* = 0.0107, respectively) in the HES group as compared to the HA group. After the second dose of colloid infusion, no further changes between the groups were detected. At the end of surgery, the following two parameters differed between the groups: CT (*p* = 0.0068) and FIBTEM-MCF (*p* = 0.0117), which registered significantly lower values in the HES test group. For Multiplate analysis, no parameters were found to be different between the two test groups.

There were no differences in the median arterial blood gas parameters nor were there any differences in median systolic, diastolic or mean arterial blood pressures, heart rate or pulse pressure variation (only measured during anaesthesia, with muscle relaxation and tidal volumes of >10 ml/kg body weight and no arrhytmias) or in intra- and post-operative median hourly diuresis between the groups. Post-operatively, there was no increase in plasma creatinine in either group (controlled 3–8 weeks postoperatively).

## Discussion

Our results indicate that the two types of colloid infusions induce coagulation defects in elective brain tumour neurosurgical patients, most notably seen with ROTEM parameters and after HES infusion. There are dose-response effects with both HA and HES. At the end of surgery, the HES group had more deranged ROTEM values than the HA group, but had been infused at a much higher volume ratio to assessed bleeding. Multiplate showed only one significant intra-group deterioration in the ADP parameter, detected after 500 ml HA, as compared to pre-surgery, and normalised already at the end of surgery. No significant Multiplate changes could be seen in the HES group.

Using viscoelastic or aggregometric techniques as point-of-care methods for assessing perioperative bleeding risks is a desirable option, since they provide faster results than traditional laboratory-based tests. ROTEM has already been used for this purpose and is able to detect colloid-induced coagulopathies (Fenger-Eriksen et al. 2009; Hartog et al. 2011); Multiplate has not been studied as thoroughly in this aspect. One study used Multiplate to detect impaired platelet function after 60 % colloid dilution in vitro (Kind et al. 2013), but the clinical implications are uncertain as this represents an extreme dilution seldom observed in clinical practice. In our study with colloid dilutions of 10–20 %, Multiplate indicated a statistically significant lowered ADP aggregation as compared to the preoperative result only after 500 ml HA. However, at all sampling points, median levels were within normal ranges for both the ADP and TRAP reagents for both fluids. It is possible that Multiplate is not sensitive enough to detect changes in platelet function at low degrees of colloid dilution in vivo. Multiplate is also affected by platelet count, especially at levels beneath 100 × 10^9^/L (Hanke et al. 2010).

Only one of our patients had a low borderline platelet count due to radiation therapy prior to surgery, and received one unit of platelet transfusion at the beginning of the surgery due to increased wound bleeding, and additionally, 250 ml HA and three units of platelets intra-operatively. This patient’s Multiplate values for TRAP/ADP were 67/30 preoperatively, 59/18 after 250 ml HA and 128/34 at the end of surgery (after the three units of platelet transfusions; patient’s data is not included in the Multiplate data statistical evaluation). This might indicate a colloid-induced effect on a low-platelet function/count that is restored after additional platelet transfusions.

In this study, blood samples were taken before the start of surgery to determine a baseline value. Some of these preoperative values fell outside of the normal ROTEM ranges, possibly induced by the tumour itself or stress due to anaesthesia (Hahnenkamp et al. 2002). After the first volume of colloid infusion, there were significant changes in several ROTEM parameters in the HES test group but almost none in the HA group. As seen in Fig. 1 and Table 2, CFT increased, and AA decreased with successive colloid infusions, which probably reflects a dilution effect on coagulation factors.

The difference in CFT and AA between test groups could be due to the different volumes of colloids given. Patients receiving HA were given an infusion of 500 ml in total, while those receiving HES had >double the volume, thus probably leading to a greater extent of initial dilution. Haemodilution could also explain why the CT parameter only changed after 1000 ml HES. The efficacy of volume replacement therapy depends on the initial plasma volume-expanding effect of the colloid and the duration of its effect. At the start of the present study, when HES data were collected, all patients received consecutive doses of 500 ml HES intra-operatively. However, with the new 5 % HA fluid regimen at our centre, very few patients received more than 500 ml for haemodynamic stabilisation and initial blood-loss replacement, probably due to a greater and enduring volume-expanding effect of HA as compared to HES (Dubniks et al. 2009). However, the fluid therapy used in this study is only empiric-based. Future studies should involve blood/plasma volume measurements or cardiac output measurements to better evaluate the colloid insult on haemostasis.

MCF measures the amplification and propagation of the clot, dependent on fibrin polymerisation and platelet function. EXTEM- and FIBTEM-MCF are the most widely used parameters in clinical settings for assessing coagulation since they correlate strongly with traditional tests, most notably plasma fibrinogen (Theusinger et al. 2013; Haas et al. 2012). In our study, EXTEM-MCF decreased after the second volume infusion of HA, but already after the first volume of HES.

With FIBTEM-MCF, we observe a similar decrease, although it was now present after the first volume of HA. This is in accordance with previous studies, since colloids are known to affect coagulation through dilution as well as interaction with coagulation factors and fibrin polymerisation. HES has been especially well-documented to exert its effects on coagulation by interacting with FXIII and fibrin polymerisation (Nielsen 2005). Fenger-Eriksen et al. showed that HES decreases coagulation factors (e.g. fibrinogen, FII, FX, FXIII) more than can be expected from haemodilution alone, suggesting that the resulting coagulopathy could be due to an acquired fibrinogen deficiency (Fenger-Eriksen et al. 2009).

The specific effects of HA on coagulation in vivo are not as well documented. Some studies imply that HA affects coagulation by decreasing platelet aggregation (Jorgensen & Stoffersen 1980; Kim et al. 1999). Another study showed that HA infusions are linked to decreased fibrinogen levels (Johnson et al. 1979); although, it is unclear whether this is merely an effect of haemodilution. Niemi et al. observed a hypercoagulative effect of HA haemodilution (Niemi & Kuitunen 1998), but this effect could not be reproduced in clinical settings (Niemi et al. 2005). In our study, the difference in volume required for MCF to be affected by each colloid might be accounted for by the different biochemical properties of HES and HA. In clinical bleeding situations, FIBTEM-MCF of <10 mm can indicate plasma fibrinogen deficiency and prompts the use of fibrinogen concentrate or fresh-frozen plasma (Bolliger et al. 2012). The minimum values were especially low in the HES group where it registered as low as 4 mm.

However, we found no correlation between lower FIBTEM-MCF values and increased perioperative bleeding in our data, as blood losses were similar in both test groups.

For inter-group comparisons, several parameters differed between the test groups after the first colloid infusion. However, after the second infusion, no further changes could be seen. Furthermore, CT and FIBTEM-MCF both differed between groups at the end of surgery. The difference in CT was already present preoperatively, but the change in FIBTEM-MCF is possibly due to the more prominent effects of HES on clot stability; as discussed earlier, HES is known to weaken clot structure (Mittermayr et al. 2007). Previous studies have found hypercoagulative ROTEM tracings at lower haemoglobin levels (Nagler et al. 2013; Solomon et al. 2013), but no such effect could be observed with certainty in our study.

At Lund University Hospital, 5 % HA is the preferred colloid for neurosurgery right now due to its ability to maintain plasma oncotic pressure. With a 20 % HA alleviation of cerebral oedema is possible (Jungner et al. 2010). However, it is hard to foresee changes in blood brain barrier that might contribute to oedema during elective brain tumour resection, so control of oncotic pressure with albumin for these types of patients is controversial.

In neurosurgery, haemostatic control poses a significant problem, as the balance between coagulation and bleeding must be maintained. If HA is to be preferred over HES, there might be a limitation to the appropriate volume of HA infusion. Either from haemodilution or another mechanism, HA seems to have an impact on coagulation. At Lund University Hospital, the upper limit of HA infusions is usually 500 ml for neurosurgical patients, with only a few receiving 750 ml. With regard to our data, infusions of these volumes might require monitoring with ROTEM or laboratory-based methods in order to detect coagulopathy and the need for fibrinogen or other transfusions. HA might be preferable in this regard since HA-induced coagulopathy is more easily reversed with fibrinogen concentrate than coagulopathy induced by HES, at least in vitro (Winstedt et al. 2013; Schlimp et al. 2013).

There are several limitations to this study. This study is not double-blinded or randomised. Initially, the aim was to study the safety of HES, as HES was our routine for haemodynamic stabilisation and initial blood-loss replacement in elective neurosurgery. This routine was later replaced by HA. It is underpowered to detect a correlation between our findings and clinical bleeding/postoperative complications. In ROTEM/Multiplate systems, results might not always correlate with clinical bleeding as there is no blood flow or endothelial interactions that affect coagulation in vivo.

We did not use the same amount of HA and HES. The patients received a mean of 375 ml HA but a mean of 861 ml HES, which is 230 % more fluid. From our very basic haemodynamic monitoring, there was an impression that the need for HA volume was much less than that of the HES volume. However, to measure this in an optimal way, we should have used blood-volume measurements or at least cardiac output monitoring. The volumes of HA and HES were not controlled by us, but by the anaesthetist in charge, trying to optimise MAP, systolic blood pressure, systolic blood pressure and PPV on top of replacing initial blood loss with 1–2 ml of the respective colloid for every millilitre of blood loss.

Dubnics M et al. have published two works on HES versus Albumin [Dubnics et al. 2007; Dubnics et al. 2009]. In short, one can say that they showed that 3 h after rescucitation with HES and HA (20 ml/kg) after bleeding in guinea pigs, plasma volume (PV) increased by 27 ml/kg in the HA group and 18 ml/kg in the HES group, equal to 1.5 times more HES needed to get the same PV-expanding effect. Corresponding PV expansion in a rat with increased permeability was 17 mL/kg for HA and 7 ml/kg of HES equal to 2.4 times as much HES albumin that needs to be given for the same PV expansion. Since the surgery, and bleeding is not likely to give the same greater permeability increase, it is more probable that the ratio for the conditions prevailing at neurosurgery are closer to 1.5 than 2.4, maybe 2.

The Saline protocol used by department may not be optimal, although we did not find any signs of hyperchloremic acidosis, hypernatremia or renal failure in our patients. Balanced crystalloids may be better [van Haren et al. 2014] from these aspects. Also, balanced crystalloids may be better than saline (and albumin) to maintain coagulation (Smith et al. 2015; Pathirana et al. 2015).

Finally, as this is a pilot study carried out during a limited period of time, the sample sizes are small, and conclusions must therefore be drawn with care regarding the preference of HA over HES as a neurosurgical fluid therapy. Nevertheless, as this study investigates clinically used fluid-therapy routines as opposed to fluid regimes designed a priori, the results are easier to apply to real-life clinical settings.

## Conclusions

There were no clinically relevant differences concerning kidney function, bleeding or coagulation; although, ROTEM and Multiplate measurements indicated both inter- and intra-group statistical differences. Albumin had a certain impact on coagulation; especially after 500 ml infusion, the ROTEM changes are close to those induced by 1000 ml hydroxyethyl starch. Clot structure measured by ROTEM FIBTEM-MCF was significantly lower with HES at the end of surgery, but HES had been infused at higher volumes to maintain intra-operative haemodynamics. Unlike previous studies that focused on thromboelastography, we also used Multiplate to assess coagulation, but no significant changes could be detected other than after 500 ml HA infusion, and those changes were normalised by the end of surgery. HA seems to be a more favourable fluid for volume replacement in neurosurgical patients at restricted volumes of infusion; however, larger studies need to be carried out for more conclusive results and preferably with plasma volume measurements. Irrespective of the type of fluid regimen, intra-operative monitoring of coagulation during neurosurgery is recommended.

## Abbreviations

AA: alpha angle
ADP: adenosine diphosphate
aPTT: activated thromboplastin time
AUS: area under curve
CFT: clot formation time
CT: clotting time
F: factor
HA: human albumin
HES: hydroxyethyl starch
MCF: maximal clot formation
PT: prothrombin time
ROTEM: rotational thromboelastometry
TRAP: thrombin receptor-activating peptide
